# LPS-matured CD11c+ bone marrow-derived dendritic cells can initiate autoimmune pathology with minimal injection site inflammation

**DOI:** 10.1177/0023677216663584

**Published:** 2016-08-03

**Authors:** Louise Saul, Dario Besusso, Richard J Mellanby

**Affiliations:** 1 Medical Research Council/University of Edinburgh Centre for Inflammation Research and Centre for Multiple Sclerosis Research, Queen's Medical Research Institute, Edinburgh, UK; 2 Royal (Dick) School of Veterinary Studies and The Roslin Institute, Division of Veterinary Clinical Studies, The University of Edinburgh, Hospital for Small Animals, Easter Bush Veterinary Centre, Roslin, Midlothian, UK

**Keywords:** refinement, mice, autoimmune, complete Freund's adjuvant, bone marrow dendritic cells, experimental autoimmune encephalomyelitis

## Abstract

The pathogenesis of human autoimmune disorders is incompletely understood. This has led to the development of numerous murine models in which the pathogenesis of autoimmunity can be probed and the efficacy of novel therapies can be tested. One of the most widely-used murine models of autoimmunity is experimental autoimmune encephalomyelitis (EAE). To induce autoimmune pathology, mice are often immunized with an autoantigen alongside an adjuvant, typically complete Freund's adjuvant (CFA). Unfortunately, CFA causes significant inflammation at the site of administration. Despite the well-recognized complication of injection site inflammation, CFA with autoantigen immunization is widely used to induce central nervous system autoimmunity. We performed a literature review which allowed us to estimate that over 10,000 mice were immunized with CFA in published EAE studies in 2013. In this study, we demonstrated that subcutaneously administered myelin basic protein (MBP)-pulsed CD11c+ bone marrow-derived dendritic cells (BMDC) were as effective at inducing EAE as subcutaneously administered MBP plus CFA. Importantly, we also discovered that the CD11c+ BMDC caused significantly less injection site inflammation than MBP plus CFA immunization. This study demonstrated that the use of CD11c+ BMDC can enable the development of autopathogenic T-cells to be studied in vivo without the unwanted side-effects of long-lasting injection site inflammation. This model represents a significant refinement to existing EAE models and may lead to the improvement of the welfare of experimental mice used to study the development of autoimmunity in vivo.

## Résumé

La pathogénèse des troubles humains auto-immuns n’est pas entièrement comprise. Cela a conduit au développement de nombreux modèles murins dans lesquels la pathogénèse de l’auto-immunité peut être prouvée et l’efficacité de nouvelles thérapies peut être testée. L’encéphalomyélite auto-immune expérimentale (EAE) est un des modèles murins d'auto-immunité les plus largement utilisés. Pour induire la pathologie auto-immune, des souris sont souvent immunisées avec un auto-antigène associé à un adjuvant, typiquement l’adjuvant complet de Freund (ACF). Malheureusement l’ACF provoque une inflammation importante au niveau du site d’injection. En dépit de la complication déjà connue de l’inflammation du site d’injection, l’ACF associé à l’immunisation auto-antigène est largement utilisée pour induire l’auto-immunité du système nerveux central. Nous avons réalisé une synthèse de la littérature scientifique qui nous a permis d’évaluer que plus de 10 000 souris avaient été immunisées avec l’ACF dans des études publiées sur l’EAE en 2013. Dans cette étude, nous avons démontré que l’injection de cellules dentritiques dérivées de la moelle osseuse (BMDC) CD11cþ pulsées avec de la protéine basique de la myéline (MBP) par voie sous-cutanée était aussi efficace pour induire l’EAE que l’association MBP plus ACF administrée par voie sous cutanée. Autre fait important, nous avons aussi découvert que les BMDDC CD11cþ provoquaient une inflammation moins importante du site d’injection que l’immunisation MBP plu ACF. Cette étude a démontré que l’utilisation de BMDC CD11cþ pouvait permettre d'étudier le développement auto-pathogène des cellules-T in vivo sans les effets secondaires indésirables de l’inflammation à long terme au site d’injection. Ce modèle représente une amélioration importante par rapport aux modèles EAE et peut conduire à une amélioration du bien-être des souris de laboratoire utilisées pour observer le développement de l’auto-immunité in vivo.

## Abstract (de)

Die Pathogenese von Autoimmunerkrankungen des Menschen ist bisher nicht vollständig geklärt. Dies führte zur Entwicklung zahlreicher Maus-Modelle, mit denen die Pathogenese von Autoimmunität untersucht und die Wirksamkeit neuartiger Therapien getestet werden kann. Eines der am meisten verwendeten Autoimmunitäts-Maus-Modelle ist experimentelle Autoimmun-Encephalomyelitis (EAE). Zur Induzierung von Autoimmun-Pathologie werden Mäuse häufig mit einem Autoantigen zusammen mit einem Adjuvans, zumeist dem kompletten Freund-Adjuvans (KFA), immunisiert. Leider verursacht KFA signifikante Entzündung an der Stelle der Verabreichung. Trotz dieser weithin bekannten Komplikation der Injektionsstellenentzündung wird KFA mit Auotantigen-Immunisierung sehr oft zur Induzierung von Autoimmunität des zentralen Nervensystems verwendet. Von uns durchgeführte Literaturstudien ermöglichten uns einzuschätzen, dass veröffentlichten EAE-Studien zufolge 2013 über 10.000 Mäuse mit KFA immunisiert wurden. In der vorliegenden Studie wiesen wir nach, dass subkutan verabreichte Myelin-Basische Protein (MBP)-gepulste aus Knochenmark abgeleitete CD11cþ dendritische Zellen (BMDC) ebenso wirksam bei der Induzierung von EAE sind wie subkutan zusammen mit KFA verabreichtes MBP. Es ist wichtig zu betonen, dass wir feststellten, dass die CD11cþ BMDC signifikant weniger Injektionsstellenentzündung verursachten als Immunisierung mit MBP und KFA. Diese Studie zeigte, dass die Verwendung von CD11cþ BMDC die Entwicklung von in-vivo zu untersuchenden autopathogenen T-Zellen ermöglichen kann ohne die unerwünschten Nebenwirkungen langanhaltender Injektionsstellenentzündung. Dieses Modell stellt eine entscheidende Verbesserung existierender EAE-Modelle dar und kann möglicherweise zu erhöhtem Wohlbefinden von Versuchsmäusen beitragen, die zu In-Vivo-Studien der Entwicklung von Autoimmunität dienen.

## Resumen

La patogénesis de los trastornos autoinmunes humanos no se conoce por completo. Esto ha llevado al desarrollo de numerosos modelos murinos en los que la patogénesis de la autoinmunidad puede explorarse y en los que la eficacia de terapias nuevas pueden probarse. Uno de los modelos murinos más utilizados de autoinmunidad es la encefalomielitis autoinmune experimental (EAE). Para inducir una patología autoinmune, normalmente se inmunizan a los ratones con un autoantígeno junto con un adyuvante, normalmente el adyuvante completo de Freund (CFA). Desafortunadamente, CFA causa una inflamación significativa en el lugar de la administración. A pesar de la bien conocida complicación de la inflamación del lugar de la inyección, la inmunización con CFA y autoantígeno es ampliamente utilizada para inducir autoinmunidad del sistema nervioso central. Llevamos a cabo una revisión de la literatura que nos permitió estimar que más de 10.000 ratones fueron inmunizados con CFA en estudios sobre EAE publicados en 2013. En este estudio, demostramos que la administración subcutánea de células dendríticas derivadas de la médula ósea CD11cþ con proteina básica de mielina [(MBP)-pulsed] fue tan eficaz para inducir EAE como la MBP administrada subcutáneamente más CFA. Asimismo, también descubrimos que CD11cþ BMDC causó una inflamación del lugar de la infección mucho menor que la inmunización con MBP más CFA. Este estudio demostró que el uso de CD11cþ BMDC puede permitir el desarrollo de células T autopatogénicas para ser estudiadas in vivo sin los efectos secundarios no deseados de la inflamación de larga duración del lugar de la inyección. Este modelo representa un refinamiento significativo de los modelos de EAE existentes y podría suponer una mejora del bienestar de los ratones de experimentación utilizados en el estudio del desarrollo de la autoinmunidad in vivo.

Autoimmune diseases, including multiple sclerosis (MS) and type I diabetes (TID), are a significant cause of morbidity and mortality. There are an estimated 540,000 individuals with MS in Europe and one million individuals with TID in USA. Collectively these diseases impose significant pain and disability on patients, shorten life expectancy, and are associated with huge direct and indirect healthcare costs.^1–3^ Autoimmune diseases present with tissue lesions suggestive of an immunopathological process, the risk genes for developing autoimmune diseases are mostly involved in modulating immune and inflammatory responses, and the most effective treatments of these diseases modulate the immune response.^4^

To further understanding of the biology of autoimmune diseases, a wide range of experimental murine models have been developed.^5,6^ One of the earliest and most widely-used autoimmune disease models is experimental autoimmune encephalomyelitis (EAE).^7^ Over 60 years ago, it was demonstrated that administration of complete Freund's adjuvant (CFA) and central nervous system (CNS) tissue could induce inflammatory lesions, which are similar to human demyelinating lesions as is typically observed in patients with MS.^7,8^ Subsequently, EAE models have played a major role in advancing understanding of immune-mediated injury and have been instrumental in the development of novel MS therapies.^7^

The diversity of EAE models has greatly expanded since the model was first described.^4,7^ The injection of CNS autoantigen responsive T-cells which have activated ex vivo is also a widely-used technique for inducing EAE.^9,10^ Spontaneous EAE models have recently been developed; and ‘humanized mice’, where genes or cells from human MS patients are transferred into murine hosts, have also been described.^4,6^ Despite these developments, the use of CFA and autoantigens remains a widely-used approach for inducing autoimmunity.^7^ One of the main advantages of the use of CFA and autoantigens for inducing autoimmunity is that this allows the development of an autopathogenic immune response to be studied in vivo, in contrast to passive transfer models which rely on the administration of ex vivo activated immune cells. However, one of the major disadvantages of using CFA in autoimmune models is that it causes long-lasting inflammation at the site of injection.^11^ Intradermal injections of CFA are associated with large palpable granulomas that often ulcerate,^12,13^ whereas subcutaneous injections can cause exudative, diffuse inflammation and fibrosis.^12,13^ In addition, mice immunized with CFA show a range of altered behaviours, including reduced open field activity and reductions in their distance travelled during forced gait analysis and voluntary wheel running.^14^ Consequently, a recent review entitled ‘Reducing suffering in EAE’ highlighted the need for careful consideration during adjuvant selection while taking into account animal welfare considerations.^15^

The disadvantages of using an adjuvant alongside autoantigens to prime naïve T-cells in vivo may potentially be overcome by administering autoantigens loaded in specialized antigen presenting cells, typically activated dendritic cells (DC).^16^ These cells express high levels of major histocompatibility complex (MHC) class II molecules and other co-stimulatory molecules and have the ability to induce proliferation and effector cytokine production from T-cells that can engage with their MHC class II and peptide molecules. However, it has proved difficult to optimize DC-driven EAE models, and only a small number of studies have reported the use of DC to initiate EAE in murine hosts.^17–19^ We have recently addressed these shortcomings by developing a novel DC-driven EAE model in which host mice, seeded with myelin basic protein (MBP) reactive T-cell receptor (TCR) transgenic CD4+ T-cells, develop a monophasic ascending paralysis following the subcutaneous administration of lipopolysaccharide (LPS)-activated, MBP-loaded bone marrow-derived DC (BMDC).^20^

Although we have demonstrated that MBP-loaded BMDC are capable of inducing EAE, it remains unclear whether our novel model avoids the complications of subcutaneously administered CFA. Consequently, the aim of this study was to address two hypotheses. The first hypothesis was that MBP-loaded BMDC and MBP plus CFA immunizations were equally effective at inducing EAE. The second hypothesis was that MBP-loaded DC would cause less injection site inflammation than CFA plus MBP immunization.

## Materials and methods

### Literature search for an annual estimate of mice immunized with CFA in EAE experiments

A PubMed search of ‘experimental autoimmune encephalomyelitis AND mice’ between 1 January 2013 and 31 December 2013 was undertaken on 27 October 2014. All abstracts were reviewed to investigate whether the study used CFA and a CNS autoantigen to induce EAE. If this information was not available in the abstract, the full paper was reviewed. In order to calculate the average number of mice used in each study, the first 30 papers published in that year were reviewed in full and the numbers of mice administered CFA were recorded.

### Mice, antigens and tissue culture medium

B10.PLxC56BL/6 (both CD45.2) and Tg4 CD45.1 mice were bred under specific pathogen-free conditions at the University of Edinburgh. All mice used in the experiments reported were female, as this allowed for standardization of experiment groups and permitted the housing of mice from different litters in the same experimental cage. The mice were maintained in individually-ventilated cages. The housing facility was compliant with the Federation of European Laboratory Animal Science Associations guidelines on screening mice for infectious diseases. Seventy-one B10.PLxC57BL/6 mice and eight Tg4 CD45.1 mice aged between six and 10 weeks were used in the experiments. All the experiments had local ethical approval from the University of Edinburgh's Animal Welfare and Ethical Review Body and were performed in accordance with UK legislation. Tg4 mice express a transgenic TCR recognizing the Ac1-9 peptide of MBP in association with I-A^u^.^21^ B10.PLxC57BL/6 mice were used as the recipient mice since immunization with CFA plus MBP or MBP-loaded BMDC does not result in disease unless the mice are pre-seeded with MBP responsive Tg4 CD45.1 CD4+ T-cells.^22^ Consequently this system provides a highly refined approach which allows the key autopathogenic T-cells to be tracked in vivo. The MBP Ac1-9(4Tyr) and Ac1-9(4Lys) peptides were obtained from Cambridge Research Biochemicals (Cleveland, UK). Tissue culture medium (RPMI 1640 medium) was supplemented with 2 mM L-glutamine, 100 U/mL penicillin, 100 µg/mL streptomycin, and 5 × 10^−5^ M 2-ME (all from Invitrogen Life Technologies, Paisley, UK) and 10% fetal calf serum (FCS; Sigma–Aldrich, Dorset, UK).

### Active induction of EAE

Female B10.PLxC57BL/6 (CD45.2) mice received 1 × 10^6^ CD4+ T-cells intravenously which had been isolated from the spleens of Tg4 CD45.1+ mice using microbeads as per manufacturer's instructions (Miltenyi Biotech, Surrey, UK). One day later (day 0), mice received 10 µg of the Ac1-9(4Tyr) peptide emulsified in CFA containing 50 µg of heat-killed *Mycobacterium tuberculosis* H37Ra (Sigma–Aldrich) at a total final volume of 100 µL, of which 50 µL was injected subcutaneously into each hind leg. On the day of immunization and 48 h later, each mouse also received 200 ng of pertussis toxin (Health Protection Agency, Dorset, UK) in 0.5 mL phosphate-buffered saline (PBS) intraperitoneally. Clinical signs of EAE were assessed daily with the following scoring system: 0, no signs; 1, flaccid tail; 2, impaired righting reflex and/or gait; 3, partial hind limb paralysis; 4, total hind limb paralysis; 5, hind limb paralysis with partial front limb paralysis; 6, moribund or dead.

### BMDC-driven EAE

BMDC were generated in the presence of recombinant granulocyte macrophage colony stimulating factor (GM–CSF; PeproTech, London, UK) for nine days, as previously described.^23^ Briefly, bone marrow was collected from the tibias of B10.PLxC57BL/6 mice, and clusters within the bone marrow suspension were dispersed by vigorous pipetting. Cells were seeded into six well plates at 2 × 10^5^/mL in 2 mL 10% FCS medium with the addition of 20 ng/mL of GM–CSF. At day 3, a further 2 mL of medium containing 20 ng/mL of GM–CSF was added to each well. At day 6, 2 mL of culture supernatant was removed and replaced with 2 mL of fresh culture medium containing 20 ng/mL of GM–CSF. To activate the BMDC, the cells were harvested at day 9 and the CD11c+ BMDC were separated by fluorescence-activated cell sorting (FACS) following staining with anti-CD11c-FITC (eBioscience, Hatfield, UK). The cells were re-plated at 2 × 10^6^ BMDC/mL with 0.1 µg/mL of LPS (Sigma–Aldrich) and 5 ng/mL of GM–CSF with 0.1 µg/mL of Ac1-9(4Tyr) for an additional 18 h.

Female B10.PLxC57BL/6 mice received 1 × 10^6^ Tg4.CD45.1 CD4+ T-cells. One day later, mice received 2 × 10^6^ LPS-stimulated, MBP (4Tyr)-pulsed CD11c+ BMDC at a total volume of 100 µL injected subcutaneously (50 µL into each hind leg). On the day of BMDC transfer and 48 h later, each mouse also received 200 ng of pertussis toxin (Health Protection Agency) in 0.5 mL of PBS intraperitoneally. Clinical signs of EAE were assessed daily as described above.

### Preparation of mononuclear cells and FACS analysis

Mice were sacrificed by CO_2_ asphyxiation, perfused with cold PBS, and mononuclear cells were prepared from the spleen and CNS as previously described.^24^ Single cell suspensions made from the spleen underwent a red blood cell lysis step using an ammonium chloride buffer (Sigma–Aldrich) and the cells were then resuspended in FACS buffer (PBS, 2% FCS, 0.01% sodium azide; Sigma Aldrich). Fc receptors were blocked with supernatant from the hybridoma 2.4G2. The antibodies used were LIVE/DEAD fixable cell stain (Life Technologies, Carlsbad, CA, USA), anti-CD4-e450 (eBioscience), anti-CD4-AF700 (eBioscience), and anti-CD45.1-Per-CP (eBioscience). For intracellular staining in response to peptides, cells were resuspended at 1 × 10^7^/mL in the presence or absence of 20 µM of 4Lys MBP. After overnight culture, 1 µL/mL of brefeldin A (eBioscience) was added for the last 4 h of culture. Cells were surface-stained prior to processing for intracellular staining using proprietary buffers according to the manufacturer's instruction (for cytokine staining; BD Biosciences, Franklin Lakes, NJ, USA). The antibodies used for intracellular cytokine staining were anti-GM–CSF–PE (eBioscience), anti-IFN–FITC (eBioscience), anti-IL-17-PerCP (eBioscience), and anti-TNF-e450 (eBioscience). FACS data were collected using LSR Fortessa (BD Biosciences) and analysed using FlowJo software (Tree Star, Olten, Switzerland).

### Grading of injection site inflammation

At necropsy, the injection site on the lateral aspect of the hind leg was examined grossly and scored using the previously reported grading system.^13^ No gross evidence of a subcutaneous swelling scored 0, 1–2 mm swellings scored 1, 2–4 mm swellings scored 2, and swellings greater than 4 mm scored 3. For histopathological evaluation, the hind legs were preserved in 4% buffered formaldehyde, decalcified, embedded in paraffin, sectioned at 5 µm and stained with haematoxylin and eosin. The histological appearance of the injection site was scored based on a previously reported grading system.^13^ The reviewer of the slides was blinded to the experimental group. Sections were taken at 500 µm intervals throughout the hind limb region and a representative slide was taken from each hind limb for scoring. No microscopic evidence of inflammation scored 0, mild inflammation scored 1, moderate inflammation scored 2, and marked to severe inflammation scored 3. The inflammation scores of the right and left legs were averaged for each mouse.

### Statistics

The peak EAE scores and inflammation scores were compared using a Mann–Whitney *U*-test. A *t*-test was used to compare the number and proportion of cells between mice which developed EAE and those which did not. A power calculation was used to calculate the number of mice used in the EAE experiment. Mice immunized with CFA typically had a peak score of 2.7 with a standard deviation of 1.6. If the peak score in the BMDC group was set at 1.1, the experiment needed a minimum number of 16 in each group to have 80% power to detect a difference between the two groups at a *P* value of < 0.05. A Fisher's exact test was undertaken to compare the proportion of mice which developed EAE following immunization with either CFA or BMDC. Significance was set at *P* < 0.05.

## Results

### Literature review of the number of mice used in EAE experiments

Initially, we estimated the number of mice administered CFA in EAE experiments by reviewing all studies which were retrieved in PubMed using the ‘experimental autoimmune encephalomyelitis AND mice’ search term that were published during 2013. This search retrieved 502 publications, of which 380 studies reported the use of CFA for inducing EAE. We reviewed 30 manuscripts in full and found that the mean number of mice per study immunized with CFA was 28 (median 23, 25th percentile 8 and 75th percentile 40). Therefore, approximately 10,640 mice were reported in scientific publications to have been immunized with CFA and autoantigen in EAE experiments in 2013.

### MBP-pulsed CD11c+ BMDC or MBP plus CFA are equally effective at initiating EAE

**Figure 1.**
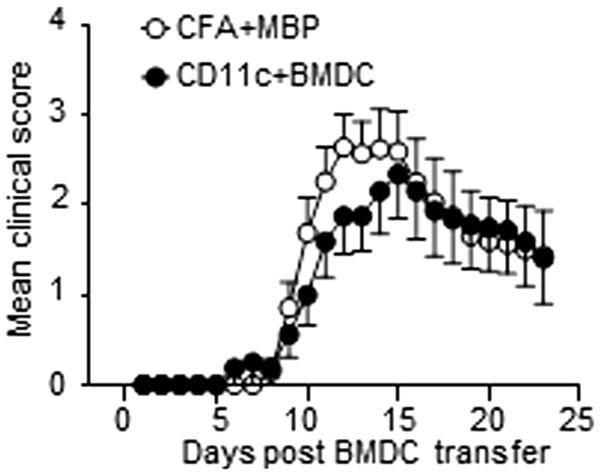
Immunization with either complete Freund's adjuvant (CFA) plus myelin basic protein (MBP) or CD11c+ bone marrow-derived dendritic cells (BMDC) is similarly effective at initiating experimental autoimmune encephalomyelitis (EAE). MBP responsive Tg4 CD4+ T-cells were transferred into B10.PLxC57BL/6 recipients on day −1. On day 0, either lipopolysaccharide (LPS)-matured, MBP-pulsed CD11c+ BMDC or MBP plus CFA were injected subcutaneously in hind limbs. Pertussis toxin was administered intraperitoneally at days 0 and 2. The clinical disease course is shown. The data shown are pooled from three independent experiments showing similar results.

We investigated our first hypothesis by examining whether our recently described BMDC-driven EAE model^20^ was as effective at inducing EAE as CFA plus MBP immunization was. In our BMDC-driven EAE model, non-transgenic host mice were first seeded with naïve CD4+ T-cells from Tg4 mice expressing a transgenic TCR recognizing the Ac1-9 peptide of MBP, prior to immunization with MBP-pulsed BMDC. We have previously shown that immunization with LPS-matured, MBP-pulsed BMDC was highly effective at inducing a robust monophasic course of EAE.^20^ In the present study, we compared the potentials of MBP-pulsed CD11c+ BMDC and MBP plus CFA immunization for inducing EAE.^20,24^ CD11c+ BMDC was used since CD11c+ expressing cells are highly effective at activating naïve T-cells.^16^ We found that both immunization protocols robustly induced EAE (Figure 1). There were no significant differences between the two groups in the proportion of mice which developed the disease (BMDC 12/16 mice, CFA 18/21, *P* = 0.44) or in the median peak severity of disease (BMDC 2.75, CFA 3.0, *P* = 0.69).

### MBP responsive T-cells accumulate in the CNS of mice which develop EAE following immunization with MBP-pulsed BMDC

**Figure 2.**
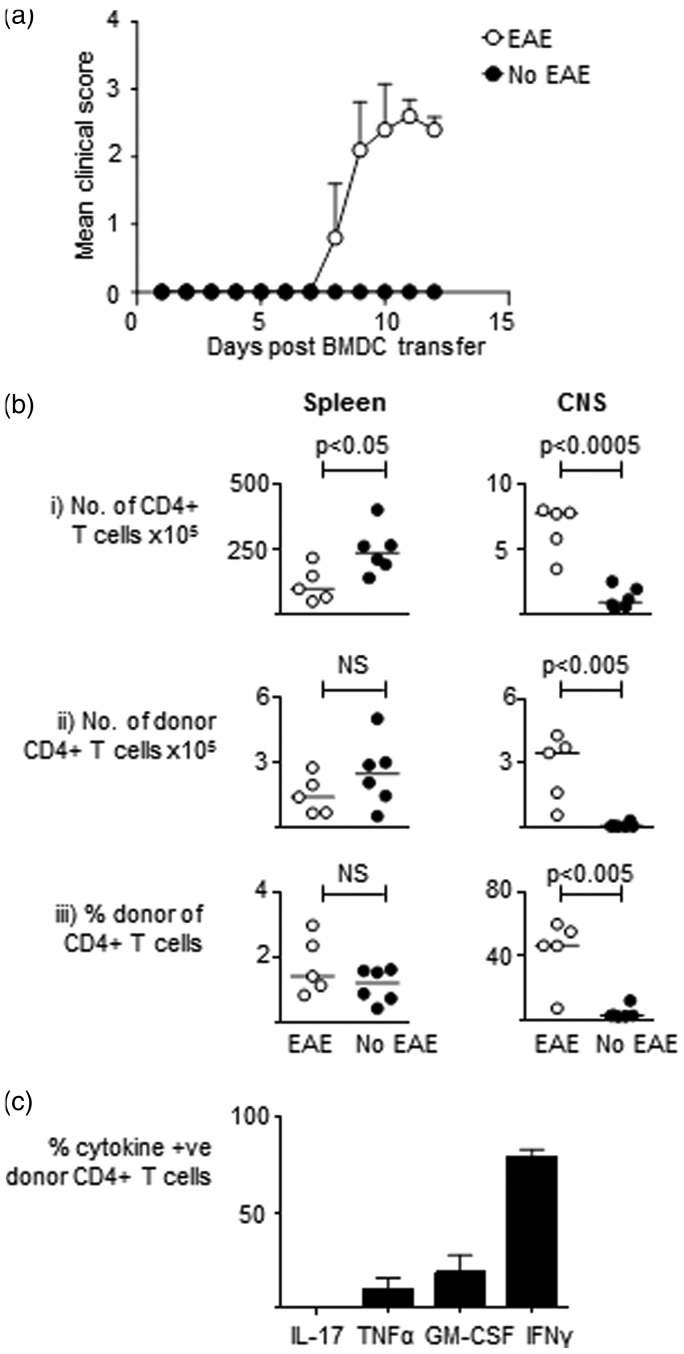
Myelin basic protein (MBP) responsive T-cells accumulate in the central nervous system (CNS) of mice which develop experimental autoimmune encephalomyelitis (EAE) following immunization with MBP-pulsed bone marrow-derived dendritic cells (BMDC). MBP responsive Tg4 CD45.1+CD4+ T-cells were transferred into B10.PLxC57BL/6 recipients on day −1. On day 0, MBP-pulsed CD11c+ BMDC were injected subcutaneously in hind limbs. Pertussis toxin was administered intraperitoneally at days 0 and 2 post CD11c+ BMDC transfer. Mice were sacrificed at day 12 and for analysis purposes they were split into mice which developed disease (*n* = 5) and those which did not (*n* = 6). (a) Disease course is shown. (b) Spleen and CNS were harvested for flow cytometry analysis of total CD4+ and donor CD4+CD45.1+ Tg4 cells. The total numbers of cells, number of donor CD4+CD45.1+ Tg4 cells, and percentage of donor CD4+CD45.1+ Tg4 cells of total CD4+ cells are shown. (c) The percentages of CD4+CD45.1+ Tg4 cells recovered from the CNS which were positive for intracellular cytokine staining following overnight stimulation with MBP are shown. Data are from one of two independent experiments containing the same number of mice giving consistent results.

The accumulation of MBP responsive autopathogenic CD4+ T-cells in the CNS of B10.PLxC57BL/6 mice which develop EAE following seeding with Tg4 cells and immunization with CFA plus MBP is well established.^25^ However, it remains unclear whether mice which develop EAE following immunization with MBP-loaded BMDC also have an increase in the number of MBP responsive T-cells within the CNS at the peak of disease. To examine this issue, we seeded B10.PLxC57BL/6 hosts with Tg4 CD4+CD45.1+ T-cells prior to immunization with MBP-loaded BMDC as per the BMDC-driven EAE protocol. We sacrificed the mice at day 12 and made single cell preparation from the spleen and CNS of all the immunized mice. For the purposes of the analysis we then split the mice into two groups based on whether they had developed EAE or had remained asymptomatic (Figure 2A). As shown in Figure 2B, there was a significant increase in total CD4+ T-cells and donor CD4+CD45.1+ Tg4 T-cells in the mice which developed disease. There was also an increase in the proportion of donor Tg4+ T-cells in the mice which developed EAE. Furthermore, we found that a significant proportion of the Tg4 CD4+ T-cells were able to produce proinflammatory cytokines such as GM–CSF and IFNγ upon re-stimulation with MBP (Figure 2C). Collectively, these findings demonstrate that the autopathogenic inflammatory infiltrate in the CNS of mice which develop EAE post MBP-loaded CD11c+ BMDC immunization is very similar to the infiltrate which has been previously reported to occur in the CNS of mice immunized with CFA plus MBP.

### MBP plus CFA immunization, but not CD11c+ BMDC, causes long-standing injection site inflammation

**Figure 3.**
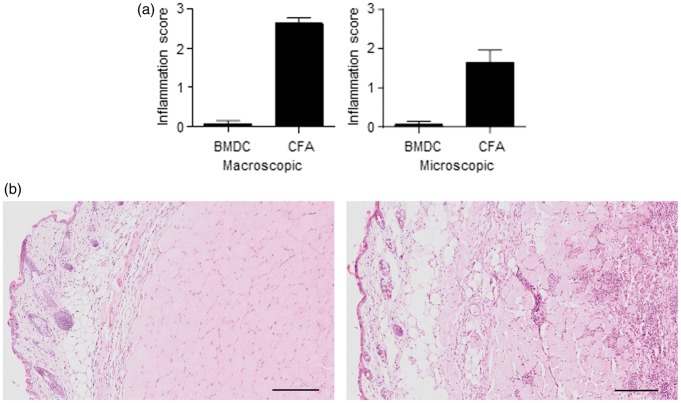
Immunization with complete Freund's adjuvant (CFA) plus myelin basic protein (MBP), but not bone marrow-derived dendritic cells (BMDC), causes injection site inflammation. Mice were immunized with either CFA plus MBP (*n* = 7) or MBP-loaded BMDC (*n* = 7) as part of the standard experimental autoimmune encephalomyelitis (EAE) induction protocol described in Figure 1. Twenty-five days post immunization, the injection sites were macroscopically examined for evidence of swelling. The injection sites were also examined microscopically for evidence of inflammation. (a) Injection site lesions were scored as detailed in Materials and methods. The histogram shows the mean value and standard error mean. (b) Representative histological examples of hind limbs from mice injected with MBP-loaded BMDC (BMDC) or CFA plus MBP (CFA) (haematoxylin and eosin stain). There is a mixed inflammatory infiltrate particularly in the subcutis and muscle of CFA plus MBP-injected mice. There is also evidence of myositis and myofiber degeneration in the CFA plus MBP-injected mice. Data are representative of two independent experiments containing the same number of mice, giving consistent results. The scale bar represents 500 µm.

Following the demonstration that CD11c+ BMDC are as effective as CFA immunization at inducing disease, we tested our second hypothesis by investigating the extent of injection site inflammation following immunization with either MBP-loaded CD11c+ BMDC or CFA plus MBP. We macroscopically and microscopically examined the injection sites 23 days post immunization in mice immunized with either CFA plus MBP or CD11c+ BMDC. All mice immunized with CFA plus MBP had a swelling at the site of injection (Figure 3A). In contrast, with the CD11c+ BMDC immunized mice, only one leg had macroscopic evidence of injection site swelling (Figure 3a) (*P* = 0.0006). Evidence of mild to moderate inflammation was routinely observed in mice immunized with CFA plus MBP, whereas only occasional mice had evidence of mild inflammation at the CD11c+ BMDC injection site (Figures 3A and B) (*P* = 0.0006). The inflammatory infiltrate in the CFA-injected mice contained a mixture of cells including macrophages, neutrophils and lymphocytes located in the subcutis and underlying musculature.

## Discussion

EAE models involving the administration of CFA and a CNS autoantigen have been instrumental in advancing understanding of the pathophysiology of autoimmune disorders and in the development of novel therapies. Their success has resulted in the development of a wide range of CFA and autoantigen-driven autoimmune models involving other body systems. These include CFA and autoantigen-driven autoimmune myocarditis,^26^ arthritis,^27^ thyroiditis,^28^ uveitis^29^ and orchitis^30^ models. Establishing the precise numbers of mice used each year in CFA-induced models of autoimmunity is difficult. Our study has estimated that over 10,000 mice had been immunized with CFA in EAE experiments which had been published in scientific papers during a single year. It is highly likely that many more mice were used in CFA-induced EAE experiments which were not reported in a published manuscript. Furthermore, large numbers of mice are immunized with CFA in non-EAE autoimmunity experiments. Therefore, our finding that several thousand mice each year are immunized with CFA to study the development of autoimmunity in vivo is likely to be a gross under-estimation of the total number.

Our study found that immunization with CFA was associated with marked localized inflammation at the site of injection. This is consistent with previous studies that have demonstrated significant pathological changes following subcutaneous injection of CFA.^11^ For example, the subcutaneous injection of CFA in mice has been shown to result in marked to severe granuloma formation, diffuse exudative inflammation and fibrosis.^13,31^ Following the subcutaneous administration of CFA, mice showed reduced movement and impaired vertical activity.^14^ In addition, mice immunized with CFA showed decreased running time on forced treadmill tests.^14^ Collectively, these behavioural changes lead to the conclusion that CFA immunization results in behavioural responses suggestive of pain.^14^

Since several thousand mice per year are immunized with CFA, which causes significant inflammation and behavioural responses suggestive of pain, there is a need to develop models which allow the in vivo development of autoimmunity to be studied without the injection site side-effects associated with CFA immunization. Previous studies have attempted to address this problem by immunizing mice with autoantigen-loaded DC rather than by immunizing with CFA and CNS autoantigen. However, none of the previously reported DC-driven EAE models have been widely employed due to a range of issues including the requirement for repeated injections of BMDC to induce EAE or the need for concurrent administration of CFA to drive robust disease.^17,19^ A previous report of MBP-pulsed DC-driven EAE differed markedly from ours in that 10 times as many MBP-reactive T-cells were transferred and the host mice were irradiated prior to cell transfer.^18^ Therefore, our study is the first demonstration, to the authors' knowledge, of a robust BMDC-driven EAE model which can initiate clinical autoimmune pathology as effectively as CFA and CNS autoantigen immunization. We have also demonstrated that in mice which develop EAE following the transfer of MBP-loaded BMDC there is an accumulation of MBP responsive T-cells which are capable of producing proinflammatory cytokines. Furthermore, we demonstrated that, in contrast to CFA immunization, BMDC immunization causes no long-term injection site inflammation. It is likely that the subcutaneously administered BMDC rapidly migrate to the draining lymph nodes where they subsequently prime the naïve Tg4 CD4+ T-cells without causing any significant subcutaneous inflammation.

A potential limitation of the BMDC-driven EAE model described in this paper is the requirement to use B10.PLxC57BL/6 hosts rather than the C57BL/6 hosts which are frequently used in EAE experiments. Immunization of C57BL/6 mice with myelin oligodendrocyte glycoprotein (MOG) is one of the most commonly used approaches for inducing EAE due to the wide availability of genetically-modified mice with a C57BL/6 background. Another potential limitation is the absence of a control group of PBS-injected mice. This was because our initial preliminary experiments demonstrated that there was very little inflammation at the injection site following administration of CD11c+ BMDC. Consequently, we felt it was ethically unjustifiable to use additional mice for a PBS injection control group when our key aim was to compare the efficacy and side-effects of CFA and BMDC injections. Despite these limitations, our model represents a significant experimental refinement which has the capacity to improve the welfare of large numbers of experimental animals.
